# A Video Decision Aid Decreases Fear of Colonoscopy After an Abnormal Fecal Immunochemical Test Result: A Pilot Study

**DOI:** 10.1007/s13187-025-02623-0

**Published:** 2025-04-05

**Authors:** Talor Hopkins, Ari Bell-Brown, Pedro Martinez-Pinto, Vida Henderson, Linda K. Ko, Anita Isler, Rachel B. Issaka

**Affiliations:** 1 Hutchinson Institute for Cancer Outcomes Research (HICOR), Fred Hutchinson Cancer Center, 1100 Fairview Ave. N., M/S: M3-B232, Seattle, WA 98109 USA; 2 Dartmouth College, Hanover, NH USA; 3 Cancer Prevention Program, Public Health Sciences Division, Fred Hutchinson Cancer Center, 1100 Fairview Ave. N., M/S: M3-B232, Seattle, WA 98109 USA; 4 Department of Health Systems and Population Health, University of Washington, Seattle, WA USA; 5 Colon Cancer Stars, Woodinville, WA USA; 6 Public Health Sciences and Clinical Research Divisions, Fred Hutchinson Cancer Center, 1100 Fairview Ave. N., M/S: M3-B232, Seattle, WA 98109 USA; 7 Division of Gastroenterology, University of Washington School of Medicine, Seattle, WA USA

**Keywords:** Colorectal Cancer Prevention, Fecal Immunochemical Test, Colonoscopy, Video Decision Aid

## Abstract

Colonoscopy completion after abnormal fecal immunochemical test (FIT) results is inadequate, and patient fear is a commonly reported barrier. We developed and piloted a video decision aid that addresses fear of colonoscopy among patients with abnormal FIT results. We developed a video decision aid and, in a pilot study, randomized patients in a safety-net healthcare system with abnormal FIT results and no follow-up colonoscopy to the intervention or usual care. Both groups completed a baseline survey that measured fear of colonoscopy, knowledge about colorectal cancer (CRC), self-efficacy, and intent to complete a colonoscopy, and the intervention group repeated the survey after watching the video. Sixty patients were enrolled in the study. Participants that watched the video reported a 17.7% decrease in fear of colonoscopy (*p* < 0.01) across six domains, including fear of the bowel prep (*p* < 0.01), the actual colonoscopy procedure (*p* < 0.01), and possible complications from the procedure (*p* = 0.04). Participant CRC knowledge also increased across several measures, including a 43.5% decrease in the belief that it is difficult to know which CRC prevention recommendations to follow. Overall, 78.3% of participants found the video to be helpful, and 90.6% would recommend the video to other patients with abnormal FIT results. In a safety-net population with abnormal FIT results, a video decision aid decreased fear of colonoscopy and increased knowledge about CRC. The video decision aid was acceptable to participants and can be considered an additional tool to improve follow-up of abnormal FIT results.

**Supplementary Information:**

The online version contains supplementary material available at 10.1007/s13187-025-02623-0.

## Introduction

Abnormal fecal immunochemical test (FIT) results must be followed by a colonoscopy to be effective [1]. Lack of follow-up colonoscopy triples the risk of being diagnosed with advanced-stage colorectal cancer (CRC) [2]. An analysis of multiple healthcare systems revealed, on average, 56% of individuals with an abnormal FIT completed a follow-up colonoscopy within 12 months [3]. Follow-up colonoscopy completion rates are even lower in safety-net healthcare settings and federally qualified healthcare centers (FQHCs) [4]. To realize the full benefit of non-invasive CRC screening strategies, there is a critical need for interventions that improve follow-up colonoscopy completion.

While multiple barriers to follow-up colonoscopy have been reported, fear of colonoscopy is critical to address due to its persistence and pervasiveness [5]. A systematic review on the association of anxiety and colonoscopy or flexible sigmoidoscopy found that 12 to 57% of patients reported moderate to severe fear of colonoscopy due to concerns about bowel preparation, procedural pain, complications, or cancer diagnosis [6]. To our knowledge, there are no interventions that have been specifically evaluated to address fear of colonoscopy after an abnormal stool-based test. Our systematic review found that interventions to improve follow-up colonoscopy in safety-net healthcare settings to date have overwhelmingly focused on the use of navigation [7]. While navigation is a promising tool, it can be time-intensive and expensive to implement. Thus, additional interventions targeting modifiable barriers, like fear, are needed to achieve the 80% U.S. Multi-Society Task Force goal for follow-up colonoscopy completion [8].

Video decision aids can improve knowledge about complicated medical procedures, improve the accuracy of risk perceptions, and decrease decision conflict, which might in turn reduce fear of colonoscopy [9]. Past studies have shown that a video delivered prior to screening colonoscopy reduced patient fear and lowered perceived procedural pain [10]. However, their effect on individuals yet to schedule a colonoscopy is unknown. We developed and tested a video decision aid for safety-net healthcare patients with an abnormal FIT needing a follow-up colonoscopy. We hypothesized that the intervention would be acceptable and would reduce fear by increasing knowledge, self-efficacy, and intent to complete a follow-up colonoscopy compared to usual care.

## Methods

### Study Design and Population

We conducted a pilot randomized controlled trial (RCT) at UW Medicine, the healthcare system affiliated with the University of Washington (UW). Participants were recruited at UW Kent Des Moines (KDM) and Harborview Medical Center (HMC), clinics that serve a safety-net population within UW Medicine. As outlined by the National Academy of Medicine, safety-net healthcare systems serve a significant proportion of vulnerable patients regardless of their ability to pay [11].

Participants were eligible if they were 45 to 75 years old, had an assigned KDM or HMC primary care provider (PCP), an abnormal FIT result for CRC screening without a follow-up colonoscopy within 3 years, and were able to communicate in English. Participants were enrolled between March 2023 and March 2024 and randomized 1:1 to either the video decision aid or usual care. Usual care varies, but actions to encourage follow-up colonoscopy after abnormal FIT results are typically managed by PCPs and are not consistently documented in the electronic health record (EHR). Therefore, our study was designed to enable analysis of the intervention’s effect on colonoscopy completion compared to usual care. Each patient received $100 compensation for their participation, and the study was approved by the Fred Hutchinson Cancer Center (FHCC) institutional review board.

### Video Decision Aid Development

The script for the video was developed in partnership with the FHCC/UW Medicine CRC screening program health equity working group, CRC survivors, and researchers with expertise in health literacy and patient education. The final video was 8 minutes long and was created in collaboration with a company experienced in creating content to address barriers to cancer screenings in medically underserved populations [12]. The intervention aimed to facilitate patient decision-making regarding colonoscopy completion by educating patients about the importance of following up after abnormal FIT results, demonstrating the steps in completing a colonoscopy, and reinforcing the implications of incomplete follow-up (Supplementary Information I).

### Survey Development

The survey included questions about social determinants of health (SDOH), fear of colonoscopy, knowledge about CRC, perceived CRC risk, self-efficacy, and intent to complete a follow-up colonoscopy. SDOH questions were derived from the Centers for Medicare & Medicaid Services health-related social needs screening tool [13]. Fear was measured by adapting Manne’s 6-item fear of colonoscopy scale [14], which assessed levels of fear associated with a colonoscopy. Items were rated on a 5-point Likert scale (1, not at all fearful; 5, extremely fearful). Knowledge and perceived risk of CRC were measured using 10 items from the Health Information National Trends Survey (HINTS) [15]. Self-efficacy was assessed by adopting an 8-item self-efficacy metric for colonoscopy completion in patients with inflammatory bowel diseases using a 10-point scale (0, I cannot do it at all; 10, Highly certain I can do) [16]. Intent to complete a colonoscopy was measured using a single validated question, and responses were dichotomized to affirmative intent (*definitely will do*, *will do)* or uncertain intent *(don’t know*, *will not do,* and *definitely will not do)* [17]. Acceptability questions were adopted from the validated Acceptability of Intervention Measure, which included four items scored on a scale of 1 to 5 (1, completely disagree; 5, completely agree). Higher scores represented greater acceptability [18].

### Study Procedures and Data Collection

Eligible participants’ contact information, assigned primary care location, date of abnormal FIT, and endoscopy details were collected from the EHR. Individuals received a mailed letter explaining the study, opt-out instructions, and up to three recruitment calls. Recruitment letters included a Quick Response (QR)-code linked to an online consent form and a phone number, if preferred, for telephonic consent. All consented participants received a survey link via text message or email and were randomized 1:1 to receive the video decision aid or usual care. Those randomized to usual care completed the survey and exited the REDCap platform. Those randomized to the intervention watched the video after the initial survey and repeated the survey post-intervention. Participants randomized to the intervention also completed a questionnaire about acceptability, impressions of the video, and viewing preferences [12].

### Data Analysis

Patient demographics, including age, gender, race, annual income, and SDOH measures were summarized as proportions or medians with interquartile ranges (IQR). Mean scores were calculated to generate average fear, knowledge, self-efficacy, and acceptability scores. We compared mean scores at baseline between usual care and intervention participants, and pre- and post-exposure to the video. Differences between groups were assessed using chi^2^ or *t*-test, as appropriate. Intent to complete a colonoscopy was analyzed using a logistic regression model. Accompanying odds ratios (OR), 95% confidence interval (CI) and *p*-values are reported in all hypothesis testing. *p*-values < 0.05 were considered statistically significant. Analyses were performed using Stata version 18.0 (StataCorp, College Station, TX).

## Results

### Participant Demographics

Ultimately, 188 eligible patients were identified, 68 (36.2%) consented, and 60 (31.9%) completed all study activities (32 randomized to intervention; 28 to usual care). The median age was 59 years (IQR 52.5–62.5), 56.7% were male, 73.3% self-identified as White, 13.3% as Hispanic, and 85.7% earned $50,000 or less annually. Those randomized to the intervention had, on average, a higher annual income than those randomized to usual care; otherwise, there were no differences between groups (Table 1). When asked about prior screening behavior, 46.4% of participants had never completed CRC screening prior to their abnormal FIT result, and 60.7% had never completed a colonoscopy.

**Table 1:** Participant demographics, *N* = 60

	Total (*N* = 60)	Usual care (*N* = 28)	Intervention (*N* = 32)	*p*-value
Age (median, IQR)	59 (52.5–62.5)	59 (53.0–68.0)	59 (52.5–62.5)	0.52
Gender				
Male	34 (56.7%)	14 (50.0%)	20 (62.5%)	0.34
Female	26 (43.3%)	14 (50.0%)	12 (37.5%)	
Race				
White	44 (73.3%)	21 (75.0%)	23 (71.9%)	0.78
Black/African American	10 (16.7%)	5 (17.9%)	5 (15.6%)	
Other^†^	6 (10.0%)	2 (7.1%)	4 (12.5%)	
Ethnicity				
Not Hispanic/Latinx	51 (85.0%)	23 (82.1%)	28 (87.5%)	0.54
Hispanic/Latinx	8 (13.3%)	4 (14.3%)	4 (12.5%)	
Unknown	1 (1.7%)	1 (3.6%)	0 (0.0%)	
Insurance				
Medicare	25 (41.7%)	13 (46.4%)	12 (37.5%)	0.36
Medicaid	23 (38.3%)	10 (35.7%)	13 (40.6%)	
Commercial	8 (13.3%)	2 (7.1%)	6 (18.8%)	
Uninsured	4 (6.7%)	3 (10.7%)	1 (3.1%)	
Relationship Status				
Single	30 (50.0%)	12 (42.9%)	18 (56.3%)	0.18
Married or common law partner	12 (20.0%)	5 (17.9%)	7 (21.9%)	
Divorced or separated	9 (15.0%)	7 (25.0%)	2 (6.2%)	
Widowed	7 (11.7%)	4 (14.3%)	3 (9.4%)	
Other*	2 (1.7%)	0 (0.0%)	2 (6.2%)	
Education Level				
Less than high school	5 (8.3%)	2 (7.1%)	3 (9.4%)	0.15
High school graduate	11 (18.3%)	4 (14.3%)	7 (21.9%)	
Training after high school	5 (8.3%)	4 (14.3%)	1 (3.1%)	
Some college or university	23 (38.3%)	14 (50.0%)	9 (28.1%)	
At least some graduate/professional training	3 (5.0%)	0 (0.0%)	3 (9.4%)	
College or university graduate	12 (20.0%)	4 (14.3%)	8 (25.0%)	
Other*	1 (1.7%)	0 (0.0%)	1 (3.1%)	
Annual Income				
< $25,000	35 (58.3%)	17 (60.7%)	18 (56.3%)	0.03
$25,000–$50,000	8 (13.3%)	7 (25.0%)	1 (3.1%)	
$51,000–$75,000	6 (10.0%)	2 (7.1%)	4 (12.5%)	
$76,000–$100,000	5 (8.3%)	1 (3.6%)	4 (12.5%)	
> $100,000	4 (6.7%)	0 (0.0%)	4 (12.5%)	
Other*	2 (3.3%)	1 (3.6%)	1 (3.1%)	
Previous CRC Screening before abnormal FIT				
No	30 (50.0%)	13 (46.4%)	17 (53.1%)	0.27
Yes	27 (45.0%)	13 (46.4%)	14 (43.8%)	
Other*	3 (5.0%)	2 (7.1%)	1 (3.1%)	
Ever completed a colonoscopy				
No	41 (68.3%)	17 (60.7%)	24 (75.0%)	0.32
Yes	16 (26.7%)	10 (35.7%)	6 (18.8%)	
Other*	3 (5.0%)	1 (3.6%)	2 (6.2%)	

*CRC* colorectal cancer, *FIT* fecal immunochemical test

^†^Asian/Asian American, American Indian/Native Alaskan, multi-racial, Native Hawaiian or other Pacific Islander

^*^Unknown or unanswered

### Fear and Knowledge

Baseline fear scores were higher in the usual care group (2.69 vs 2.30). Among intervention participants who completed both a pre- and post-video survey (*n* = 29), the mean fear of colonoscopy score decreased by 17.7% (1.90 vs 2.30, *p* < 0.01) after exposure to the video. Fear decreased across multiple domains, including fear of the colonoscopy prep (22.2%, *p* < 0.01), fear of the actual procedure (18.9%, *p* < 0.01), and fear of the procedure being painful (24.1%, *p* < 0.01) (Table 2). Participant knowledge also improved across multiple domains in the intervention group. Questions related to knowledge are summarized in Table 3.

**Table 2:** Survey results—fear of colonoscopy

**Survey questions**	**Mean scores**				
Please rate how fearful you are about the following aspects of the colonoscopy procedure. * How fearful are you of…					
	Pre-video^†^	Post-video^†^	Difference (%)	95% CI	p-value
The overall colonoscopy procedure	2.31	2.10	− 0.21 (9.1%)	− 0.53, 0.12	0.10
The colonoscopy prep	2.34	1.83	− 0.52 (22.2%)	− 0.92, − 1.12	< 0.01
The actual colonoscopy procedure	2.33	1.89	− 0.44 (18.9%)	− 0.76, − 0.13	< 0.01
The procedure being painful	2.45	1.86	− 0.59 (24.1%)	− 0.88, − 0.29	< 0.01
Possible complications from procedure	2.48	2.10	− 0.38 (15.3%)	− 0.82, 0.06	0.04
Having to tell your family about results	1.86	1.52	− 0.34 (18.3%)	− 0.65, − 0.03	0.02
Overall mean fear score	2.30	1.90	− 0.41 (17.7%)	− 0.61, − 0.22	< 0.01
Is thinking about CRC emotionally stressful?	2.14	1.96	− 0.18 (8.4%)	− 0.33, − 0.03	0.01

*CRC* colorectal cancer

^†^Includes the 29 out of 32 patients who completed both the pre- and post-video survey

^*^5-point scale. 1 = “Not at all Fearful”; 5 = “Extremely Fearful”

**Table 3:** Survey results—CRC knowledge, *N* = 60

Question	*N* (%)		
	Usual care (*N* = 28)	Pre-video (*N* = 32)	Post-video (*N* = 32)
There's not much you can do to lower your chances of getting colon cancer			
Agree	10 (35.7%)	10 (31.3%)	8 (25.0%)
Disagree	18 (64.3%)	20 (62.5%)	22 (68.8%)
Not answered	0 (0.0%)	2 (6.3%)	2 (6.3%)
There are so many different recommendations about preventing colon cancer that it's hard to know which ones to follow			
Agree	21 (75.0%)	22 (71.9%)	13 (40.6%)
Disagree	7 (25.0%)	8 (25.0%)	17 (53.1%)
Not answered	0 (0.0%)	2 (3.1%)	2 (6.3%)
Colon cancer develops over a period of several years			
Agree	21 (75.0%)	23 (71.9%)	22 (68.8%)
Disagree	5 (17.9%)	8 (25.0%)	8 (25.0%)
Not answered	2 (7.1%)	1 (3.1%)	2 (6.2%)
There are ways to slow down or disrupt the development of colon cancer			
Agree	22 (78.6%)	25 (78.1%)	27 (84.4%)
Disagree	6 (21.4%)	6 (18.8%)	3 (9.4%)
Not answered	0 (0.0%)	1 (3.1%)	2 (6.2%)
Colon cancer is most often caused by a person's behavior or lifestyle			
Agree	8 (28.6%)	9 (28.1%)	13 (40.6%)
Disagree	20 (71.4%)	22 (68.8%)	17 (53.1%)
Not answered	0 (0.0%)	1 (3.1%)	2 (6.3%)
It seems like almost everything causes colon cancer			
Agree	6 (21.4%)	5 (15.6%)	7 (21.9%)
Disagree	21 (75.0%)	26 (81.3%)	23 (71.9%)
Not answered	1 (3.6%)	1 (3.1%)	2 (6.2%)
You are reluctant to get checked for colon cancer because you fear you may have it			
Agree	10 (35.7%)	7 (21.9%)	6 (18.8%)
Disagree	17 (60.7%)	24 (75.0%)	24 (75.0%)
Not answered	1 (3.6%)	1 (3.1%)	2 (6.2%)
Getting checked regularly for colon cancer increases the chances of finding cancer when it's easy to treat			
Agree	25 (89.3%)	28 (87.5%)	28 (87.5%)
Disagree	3 (10.7%)	3 (9.4%)	2 (6.2%)
Not answered	0 (0.0%)	1 (3.1%)	2 (6.2%)
People with colon cancer would have pain or other symptoms prior to being diagnosed			
Agree	10 (35.7%)	12 (37.5%)	5 (15.6%)
Disagree	18 (64.3%)	18 (56.3%)	25 (78.1%)
Not answered	0 (0.0%)	2 (6.2%)	2 (6.2%)

### Self-Efficacy and Intent to Complete a Colonoscopy

Across all measures, the mean self-efficacy score increased after exposure to the intervention (7.24 to 7.76, *p* = 0.02), with significant increases in the ability of participants to accept the news that it was time for a colonoscopy, to schedule or reschedule colonoscopy appointments, and to tolerate the colon cleaning process.

Baseline intent to complete a colonoscopy was higher in those randomized to the intervention prior to viewing the video compared to usual care (71.9% vs. 53.6%, OR 2.21, 95% CI 0.76, 6.46; *p* = 0.15). After watching the video, uncertain or negative intent to complete a colonoscopy decreased by 50.0% in those randomized to the intervention (OR 0.45, 95% CI 0.15, 1.32; *p* = 0.15). There were no associations between patient-level factors (sex, race, ethnicity, etc.) or prior CRC screening behaviors and intent to complete a follow-up colonoscopy.

### Acceptability

Across all acceptability measures, the mean acceptability score was 3.75 out of 5. Most participants approved of the video (4.00) and found it appealing (3.70). The majority (*n* = 25/32, 78.1%) reported increased understanding of colonoscopy importance and that they would recommend it to others with an abnormal FIT result.

### Follow-Up Colonoscopy Completion

While this pilot study was inadequately powered to evaluate changes in follow-up colonoscopy completion, a preliminary analysis notes a trend towards increased 6-month colonoscopy completion in those randomized to the intervention vs usual care (38% vs 36%, *p* = NS).

## Discussion

Fear of colonoscopy is a persistent barrier to follow-up colonoscopy; yet, few interventions address this issue. This pilot study found that a low-health literacy video decision aid reduced fear of colonoscopy completion after an abnormal screening result, increased knowledge about CRC, and intent to complete a follow-up colonoscopy, and was acceptable to most participants. These findings suggest that offering video decision aids might be one strategy to address the patient-level barrier of colonoscopy fear, as it pertains to follow-up of non-invasive CRC screening tests.

Completing a follow-up colonoscopy is complex and includes understanding the implications of the abnormal screening result, completing the bowel preparation, the procedure itself, which might include sedation, and understanding the results and follow-up. This process might also differ depending on SDOH and healthcare team relationships. In our video, the main character is counseled about her abnormal FIT results and the need for a follow-up colonoscopy by her doctor. She completes the bowel prep process and colonoscopy procedure and receives her results, which she discusses with friends and family; each scene is intended to address an aspect of colonoscopy fear. For these reasons, it is conceivable that a video would reduce overall fear of follow-up colonoscopy completion. In a 2010 study that compared verbal descriptions of advanced dementia to a video that showed someone with advanced dementia, researchers found that viewing a video led to deeper patient engagement by enabling participants to put themselves “in the shoes” of the person in the video [19]. After viewing our video, study participants closely aligned themselves with the attitudes and beliefs of our video’s main character, which provides a mechanism for the observed results. Visual information also enables patients to envision contexts and outcomes, potentially reducing the mystery— and therefore reducing fear— surrounding the colonoscopy process [20].

Like prior studies, we found that a video decision aid increased participants’ knowledge [21]. After watching the video, participants had a better understanding of the recommended tests for CRC screening, which might influence follow-up of abnormal results, learned that they could change their risk of being diagnosed with CRC, and that CRC did not always present with pain. Additionally, participants were more intent to complete a follow-up colonoscopy, which might be due to a strategy called “video modeling” [22]. In our video, the main character models each step of the follow-up colonoscopy process, including discussing the procedure with her doctor, undergoing bowel prep, receiving the colonoscopy— which includes real footage from a colonoscopy procedure— and interpreting her results. This strategy of visually demonstrating desired behaviors has been shown to increase self-care behaviors in numerous studies [23].

Our video was highly acceptable to a safety-net population with abnormal FIT results. Participants found the video appealing and felt comfortable viewing it. This is potentially due to increased use of videos as a means of communication over the last 20 years, rising familiarity with smartphones to access videos in other spheres, and the convenience of videos [24, 25]. In the U.S., over 76% of adults ≥ 50 years old, which comprises most of the eligible screening population, owned a smartphone in 2023. Additionally, research supports videos as effective tools for relaying health information to broad audiences because they do not depend on reading comprehension. Video decision aids are also a potentially scalable intervention. A single video, once produced, can be distributed widely and used indefinitely, at little to no cost.

Our study had limitations. First, our results should be interpreted in the context of the trial design. Most trial participants were insured through a government insurance program, earned less than $50,000/year, and had never completed a colonoscopy. While this is relevant for other safety-net settings, future work could include adaptations for non-safety-net populations. Second, individuals randomized to usual care who were not exposed to the intervention reported higher social needs and higher baseline fear of colonoscopy scores compared to those randomized to the intervention, which might have potentially led to underreporting of the intervention effect in our pilot study. We also recognize that usual care varies considerably across providers and health systems. Within our health system, there was increased recognition about the importance of follow-up colonoscopy, in part due to messaging from a centralized CRC screening program. Third, the video was delivered via a REDCap study platform; real-world use of the video decision aid accessed in clinical and non-clinical spaces is an important target of our future work. Finally, as this was a pilot, adequately powered future studies are needed to examine the association between the video, reduced fear, and follow-up colonoscopy completion and persistence of the reduction in fear across groups.

Our study also had several strengths. This trial demonstrated that a video decision aid focused on the colonoscopy procedure was acceptable in a safety-net population. This is an ideal population for such an intervention, given a preference for non-invasive CRC screening strategies and suboptimal follow-up colonoscopy completion. Video decision aids are relatively easy for both patients and health systems; they can be easily embedded in existing EHR platforms and are generally inexpensive to distribute, creating the potential for extensive reach. Our findings set the foundation for efficacy trials that examine the association between video decision aids, fear, and ultimately colonoscopy completion after abnormal FIT or other non-invasive CRC screening tests. While it is unlikely that a single intervention can address the challenge of inadequate follow-up, future studies could evaluate the role of a video decision aid as part of multilevel interventions to address this issue.

In conclusion, in a pilot RCT, we found that a video decision aid decreased fear of colonoscopy, increased knowledge about CRC, increased intent to complete a colonoscopy, and was acceptable to participants from a safety-net health system population. Our results demonstrate that on-demand, point-of-care videos offer a potentially scalable and cost-effective strategy to improve follow-up colonoscopy rates after non-invasive CRC screening tests, such as FIT, and could be incorporated into multilevel strategies to improve overall CRC screening participation.

## Supplementary Information

Below is the link to the electronic supplementary material.

Supplementary file1 (DOCX 32 KB)
