# Supplementary material for: A Video Decision Aid Decreases Fear of Colonoscopy After an Abnormal Fecal Immunochemical Test Result: A Pilot Study
Source: J Cancer Educ. 2025 Apr 5;41(1):55–62. doi: 10.1007/s13187-025-02623-0 (PMC12971742; doi:10.1007/s13187-025-02623-0)

Supplementary Information I:  Video Decision Aid Clip

Scan the code below or visit **www.bit.ly/3OajTmI** to watch a 1-minute clip of our video decision aid.


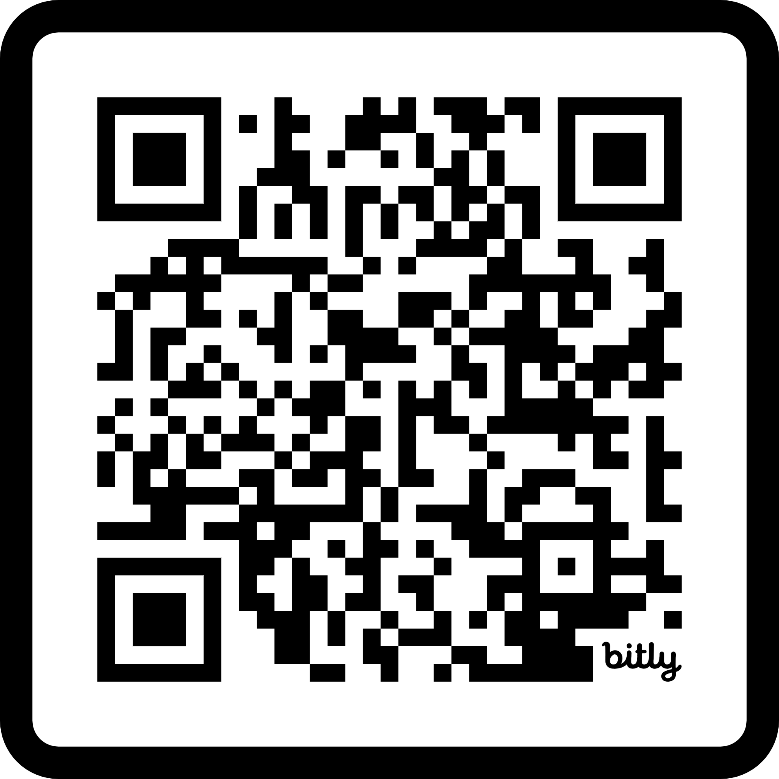

Supplement: Supplementary file 1 — Supplementary file1 (DOCX 32 KB) [file 13187_2025_2623_MOESM1_ESM.docx]
